# Repurposing FDA-approved drugs as FXR agonists: a structure based *in silico* pharmacological study

**DOI:** 10.1042/BSR20212791

**Published:** 2023-03-01

**Authors:** Sandra Jose, Sreevidya S. Devi, Anjana Sajeev, Sosmitha Girisa, Mohammed S. Alqahtani, Mohamed Abbas, Abdulrahman Alshammari, Gautam Sethi, Ajaikumar B. Kunnumakkara

**Affiliations:** 1 Cancer Biology Laboratory, Department of Biosciences and Bioengineering, Indian Institute of Technology (IIT) Guwahati, Guwahati, Assam 781039, India; 2 Radiological Sciences Department, College of Applied Medical Sciences, King Khalid University, Abha 61421, Saudi Arabia; 3 BioImaging Unit, Space Research Centre, Michael Atiyah Building, University of Leicester, Leicester, LE1 7RH, U.K.; 4 Electrical Engineering Department, College of Engineering, King Khalid University, Abha 61421, Saudi Arabia; 5 Computers and Communications Department, College of Engineering, Delta University for Science and Technology, Gamasa 35712, Egypt; 6 Department of Pharmacology and Toxicology, College of Pharmacy, King Saud University, Post Box 2455, Riyadh 11451, Saudi Arabia; 7 Department of Pharmacology, Yong Loo Lin School of Medicine, National University of Singapore, Singapore; 8 NUS Centre for Cancer Research (N2CR), Yong Loo Lin School of Medicine, National University of Singapore, Singapore

**Keywords:** agonists, FXR activators, molecular docking, molecular dynamics simulations

## Abstract

Farnesoid X receptor (FXR) modulates the expression of genes involved in lipid and carbohydrate homeostasis and inflammatory processes. This nuclear receptor is likely a tumor suppressor in several cancers, but its molecular mechanism of suppression is still under study. Several studies reported that FXR agonism increases the survival of colorectal, biliary tract, and liver cancer patients. In addition, FXR expression was shown to be down-regulated in many diseases such as obesity, irritable bowel syndrome, glomerular inflammation, diabetes, proteinuria, and ulcerative colitis. Therefore, development of novel FXR agonists may have significant potential in the prevention and treatment of these diseases. In this scenario, computer-aided drug design procedures can be resourcefully applied for the rapid identification of promising drug candidates. In the present study, we applied the molecular docking method in conjunction with molecular dynamics (MD) simulations to find out potential agonists for FXR based on structural similarity with the drug that is currently used as FXR agonist, obeticholic acid. Our results showed that alvimopan and montelukast could be used as potent FXR activators and outperform the binding affinity of obeticholic acid by forming stable conformation with the protein *in silico*. However, further investigational studies and validations of the selected drugs are essential to figure out their suitability for preclinical and clinical trials.

## Introduction

Farnesoid X receptor (FXR) α1, also called as bile acid receptor has a role in cholesterol, glycogen, and energy balance [1,2]. FXR (gene name: NR1H4) plays a vital role in lipid and glucose metabolism, cellular differentiation and proliferation, inflammation, liver regeneration and fibrosis, and is a member of the nuclear receptor superfamily [1–5]. FXR is highly expressed in tissues of heart, liver, adipose tissue, intestine, blood vessels, kidneys, and adrenal glands [2,5,6]. Activated by bile acids and a metabolic intermediate called as farnesol, this receptor binds to the DNA–FXR response elements with its common partner, namely the retinoid X receptor (RXR) to modulate the expression of several genes associated with the breakdown of glucose and lipids. Thus, FXR is primarily controlled by the levels of bile acid and henceforth panels the bile acid metabolism, absorption, and transport [6,7]. However, alteration in the FXR expression might lead to severe health complications like liver injury, cholestasis, gastrointestinal (GI) disorders, and mucosal injury [8]. Studies of obesity and gut microbiome improvement showed that FXR agonism modulated body mass index, irritable bowel syndrome and ulcerative colitis [9–14]. The dyslipidemic phenotype of FXR null mice is indicative of FXR's crucial function in cholesterol and glycemic homeostasis [15]. Synthetic FXR agonists have now been developed to stimulate this receptor particularly for treatment of renal lipid accumulation and non-alcoholic liver steatohepatitis (NASH) [16]. Several studies targeted the regulation of renal signaling pathways that are controlled by FXR for diabetes and glomerular inflammation treatment [17–19]. The role of FXR can possibly be a tumor suppressor gene or an oncogene, depending on the cell and tissue type [8,20,21]. Several studies reported that decreased FXR expression leads to tumorigenic phenotypes in breast, bile duct, colon, liver, and intestine while there are also studies that support that the overexpression of FXR protein induces non-small cell lung cancer and esophageal cancer and esophageal adenocarcinoma [20,22–28].

Recently, studies also suggest that FXR downregulation in cancer cell lines promoted cellular proliferation indicating that FXR activation can be a potential therapeutic option for the treatment of these cancers [25–28]. In addition to its involvement in cancer suppression, FXR also plays an important role in the differentiation of stem cells into functional hepatocytes or intestinal cells and henceforth is the master regulator of bile acid synthesis, homeostasis, detoxification processes and flow throughout the gut–liver axis [28–31]. It is reported that bile acid derivatives have the property to induce the expression of FXR and hence control tumorigenesis, glomerular inflammation and tubulointerstitial fibrosis [32,33].

Besides bile acid derivates such as cholic acid, deoxycholic acid, chenodeoxycholic acid, lithocholic acid, and several agonists of FXR have been reported like GW4064, AGN31, and 6-ethylchenodeoxycholic acid [34–36]. GW4064, the first nonsteroidal and selective agonist of FXR is studied to lower the free-fatty acid and triglyceride levels and is used as a structural template for identification of novel FXR agonists [34]. Recently, several *in silico* and *in vitro* experiments reported novel potential agonists of FXR through high-throughput screening [37–39]. For example, Grienke et al. explored the agonistic potential of triterpenes in *Ganoderma lucidum* Karst. as FXR ligands [40]. This study revealed the stimulation of FXR in low micromolar range using compounds such as ergosterol peroxide, ganoderiol F, ganoderic acid TR, and lucidumol A [40]. Preclinical and clinical investigations reported tropifexor (LJN452) and nidufexor (LMB763) as potent FXR agonists; most potent being tropifexor while in individuals with NASH and nephropathy, nidufexor has moved to phase II clinical trials [41–43]. Obeticholic acid is a food and drugs administration (FDA) approved drug for the treatment of fibrosis which is caused by NASH. It is a bile acid analog, also used in the primary biliary cholangitis treatment in adult patients with inadequate clinical response or intolerance to ursodeoxycholic acid (UDCA) [44–47]. However, treatment with obeticholic acid reports several side effects that consist of macular eruption, extreme papular rash, several cases of urticaria, and severe pruritus at different locations including, eye, ears, and anal region [48,49]. Hence, there is an urgent need to find potential drugs that can function as FXR agonists and computer-aided drug repurposing of FDA-approved drugs would ease the discovery by reducing time and cost. In this light, we discuss the potential of FDA-approved drugs in controlling the expression of FXR using *in silico* techniques. These drugs were selected based on their similarity to the structure of obeticholic acid and examined for their binding affinity to the target protein. The procedure used in this *in silico* analysis is virtual screening accompanied by molecular dynamics (MD) simulation and trajectory analysis that enhances the pace of conventional *in vitro* procedures. This way a large selection of molecules could be analyzed in a very short period in a cost-efficient way. This is significantly helpful during a pandemic when there is enormous pressure on scientists to formulate novel medicines quickly even when situations are not favorable.

Hence, in the present study, we have performed molecular docking-based virtual screening for 109 FDA-approved drugs from the PubChem database, MD simulations, absorption, distribution, metabolism, excretion, and toxicity (ADMET) analysis of the drugs to examine how different FDA-approved drugs interact with the FXR receptor while also finding the best drugs from the whole-sum that would form the complex with the highest affinity between the protein and drug. Further, MD simulations results were analyzed by different parameters root mean square fluctuation (RMSF), solvent accessible surface area (SASA), radius of gyration (Rg), interaction energy, and principal component analysis (PCA). Therefore, the present study was undertaken to analyze the behavior of the complex using the protein–ligand complex against FXR.

## Materials and methods

### Preparation of the ligands

The structural similarity to the FDA-approved drug, obeticholic acid was used as the base for the generating a focused library containing FDA-approved drugs with structural similarity score >0.004. The structural similarity scores were determined using Swiss Similarity (http://www.swisssimilarity.ch/) and the 3D structures of the drugs were downloaded from the PubChem database (https://pubchem.ncbi.nlm.nih.gov) in the .sdf format [50–52]. In total, the library consists of 110 drugs including the control. These .sdf files were converted into .pdb files using PyMol software [53]. These structures were subjected to molecular docking using AutoDock Tools [54]. The preparation and docking were done using the standard protocol and the .pdbqt files were saved.

### Preparation of FXR

**Figure 1:**
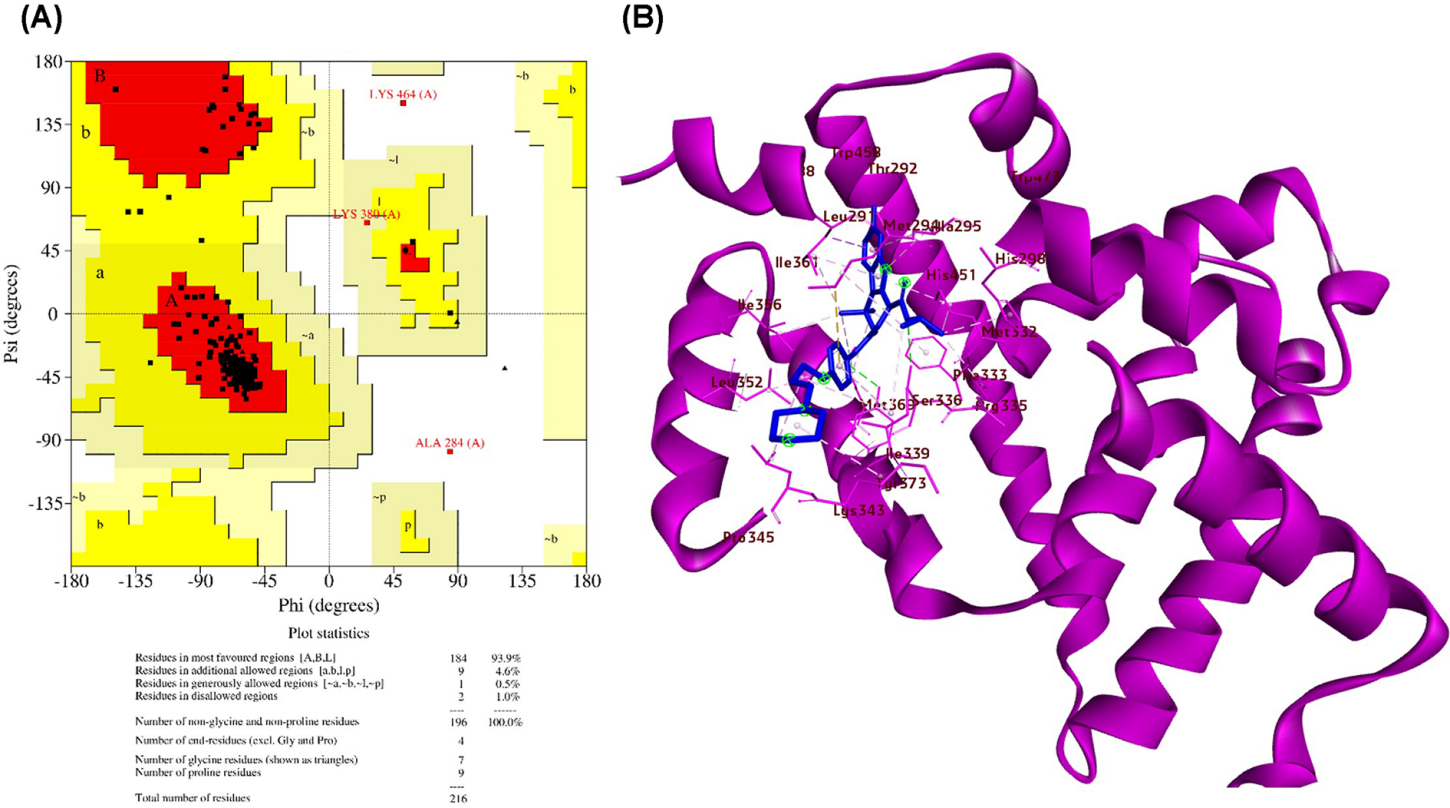
Structural details of FXR (**A**) Ramachandran plot analysis of FXR determined by PROCHECK. Region in red of the plot indicates most favored region (93.9%), while the yellow region denotes the additionally allowed region (4.6%). The light yellow region in the plot accommodates the generously allowed amino acids (0.5%) and the white region is the disallowed region where two amino acid residues lie (1.0%). (**B**) The structure of FXR, PDB ID: 3L1B with the native ligand. Tetrahydroazepinoindole (in blue) depicts the interacting amino acids (sticks representation) with the protein which is represented in a purple ribbon structure. The image is prepared using Discovery Studio3.5.

The complex structure of FXR ligand-binding domain with a tetrahydroazepinoindole compound was downloaded from protein databank (PDB) (3L1B) (PDB ID: 3L1B) as represented in Figure 1B [55]. The quality of the .pdb file was assessed by plotting the Ramachandran plot using the PROCHECK module of SAVES 6.0. and is represented in Figure 1A, and the structure reported acceptable values for Ramachandran most favored region (93.9%) [56,57]. The file was checked for side chain abnormalities, improper bond angles and these were rectified. The .pdb structure was prepared as the macromolecule for docking using MGL tools as per the standard procedures and .pdbqt file was saved.

### Molecular docking

The molecular docking of the FXR was performed with the drugs of the prepared focused library saved in their .pdbqt file formats using AutoDock Vina. During the process of molecular docking, the solvent molecules were removed and polar hydrogen and Kollman’s charges were added to the protein structure. Further, the number of point was set to 40 in the three dimensions (*x, y*, and *z*) and 0.375 Å spacing. The configuration was set with an energy range of 4 and exhaustiveness to 8. The log files obtained after every run were saved as log.txt file. The binding affinity values which were obtained from Autodock Vina were analyzed to obtain the best drugs which showed the highest binding affinities (< −7.0 Kcal/mol). The active site of the protein, that is, structure of ligand binding site of the protein is available in the PDB, with active ligand as tetrahydroazepinoindole compound which is used as the reference for optimized docking process. Hence, the residues that form the active site were analyzed critically and the grid box was centered at *x* = −5.0114, *y* = −7.5753, *z* = 14.8261 with dimensions, *X*: 35.3378336811, *Y*: 48.5275607109, and *Z*: 58.7552267742, which was a suitable grid box volume where the ligands have enough flexibility to fit anywhere inside the entire active site region. To compare the activity of the drugs, the affinity energy values were compared with the control, obeticholic acid which is currently the FDA-approved drug used as an FXR agonist. The control drug was also docked in the same protocol as for the other drugs. For each drug, different docked poses were obtained with different root mean square deviation (RMSD) values. The docked pose with the least RMSD was selected to calculate the binding affinity between the FXR and the drugs of the library. The output was visualized using PyMol and Discovery Studio 3.5 [58].

### ADMET analysis

ADMET analysis was performed for all the drugs in the library which showed the highest affinity (docking energy equal to or less than −8.4 Kcal/mol) to examine them for their absorption, distribution, metabolism, excretion, and toxicity characteristics. This study was performed using SWISS ADME (http://www.swissadme.ch/) and PreADMET (https://preadmet.qsarhub.com/) and the results were saved in the .csv format and analyzed [59].

### Molecular dynamics simulation

The MD simulation was performed for the apo-protein and the protein in a bound state with the drugs chosen for MD, as well as the control. GROningen MAchine for Chemical Simulations (GROMACS) version 5.1.2 was used to run the MD simulations, which used the optimized potentials for liquid simulations-all atom/LMP2 (OPLS-AA/L) forcefield and transferable intermolecular potential with 3 points (TIP3P) water model (TIP4P) [60,61]. The TIP3P or TIP4P water model is commonly used in OPLS simulations in aqueous solution. The OPLS parameters were optimized to fit experimental properties of liquids, such as density and heat of vaporization, as well as gas-phase torsional profiles [60]. The FXR topology files were created with GROMACS, while the ligand topology files were created with the Jorgensen group’s LigParGen server, which is a web-based service that provides force field parameters, most notably the .itp and .gro files for ligands. With 1.14*CM1A or 1.14*CM1A- LBCC partial atomic charges, LigParGen provides bond, angle, dihedral, and Lennard-Jones OPLS-AA parameters. The generated .itp and .gro files were used in further MD simulation steps [62]. The provided water model extends 10 Å beyond all of the atoms in the protein–ligand complex. Initially, the complex’s total charge was calculated as −11.000 e, thus 11 solute molecules in the topology file were replaced with 11 NA and 0 CL ions, resulting in a total number of solute molecules in the system of 14906.

The LINear Constraint Solver (LINCS) algorithm was used to constrain all protein bond lengths, whereas the SETTLE algorithm was used to restrain water molecules [61]. Each system was energy-minimized and equilibrated to achieve the desired volume using the steepest descent algorithm. A cutoff of 0.1 nm was used to compute short- and long-range nonbonded interactions. The equation of motion was integrated using the leapfrog algorithm with a time step of 2 fs. The Particle Mesh Ewald method is used to treat long-range electrostatics, using a Fourier grid spacing of 0.16. In all three directions, periodic boundary conditions were applied.

For 10 ns, the system was allowed to gradually heat to 300 K in a NVT [number of particles (N), volume (V), temperature (T)] ensemble using the velocity-rescale algorithm [60]. The complexes were next restrained for 5 ns as the solvent molecules gradually relaxed around them and then another 10 ns of NPT [number of particles (N), pressure (P), temperature (T)] equilibration was performed by gradually removing the restraints on the complexes. For temperature coupling, we applied modified Berendsen thermostat (two coupling groups) and a Parrinello-Rahman barostat (isotropic). The average temperature and pressure readings for each system remained close to the desired values [63]. The equilibrated systems were then subjected to 100 ns unrestrained production MD simulations by maintaining target pressure (1 bar) and temperature (300 K). Every 10 ps, the energy and updated log file were saved. Finally, GROMACS modules such as gmx rms, gmx rmsf, gmx gyrate, and gmx hbond were used to calculate RMSD, RMSF, *R*_g_, and hydrogen bond analysis for MD trajectories. The gmx sasa, which measures the receptor area exposure to the solvents during the 100 ns simulation process, was used to calculate the total SASA.

### Principal component analysis

The idea of bioactive molecules, which is described as a multifaceted space representation of the number of property characteristics derived for each chemical entity, has now become crucial in the process of drug discovery. PCA is a technique for identifying and highlighting prevalent patterns of drug-like substances by visualizing chemical space in lower dimensions. PCA reduces the dataset’s dimensions and improves comprehensibility and the estimates were obtained using gmx anaeig and gmx covar [64].

## Results

### Site-directed molecular docking

**Figure 2:**
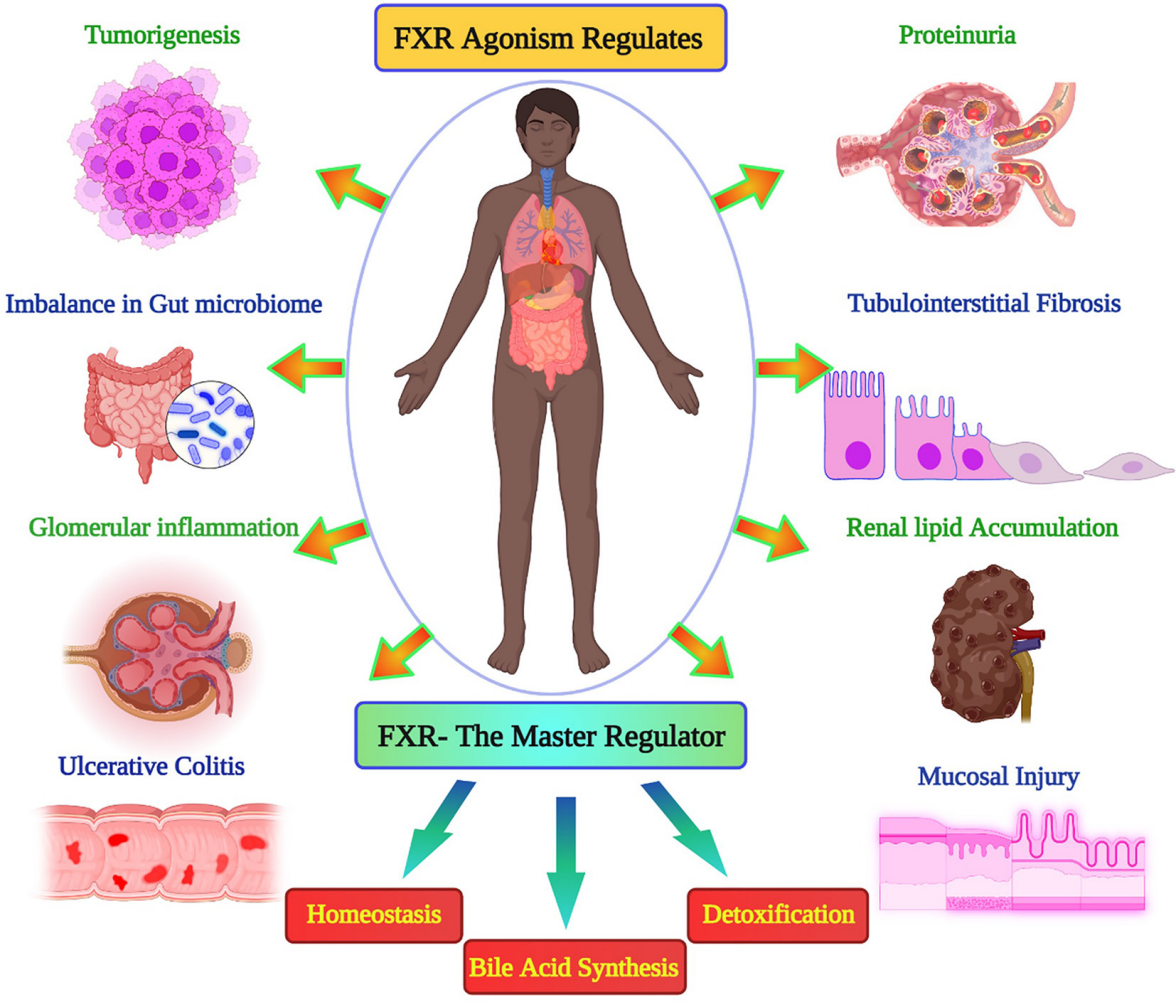
Important roles of FXR Depiction of role of FXR in various health conditions such as tumorigenesis, imbalance in gut microbiome, tubulointerstitial fibrosis while FXR is also the master regulator of homeostasis, bile acid synthesis, and detoxification.

**Figure 3:**
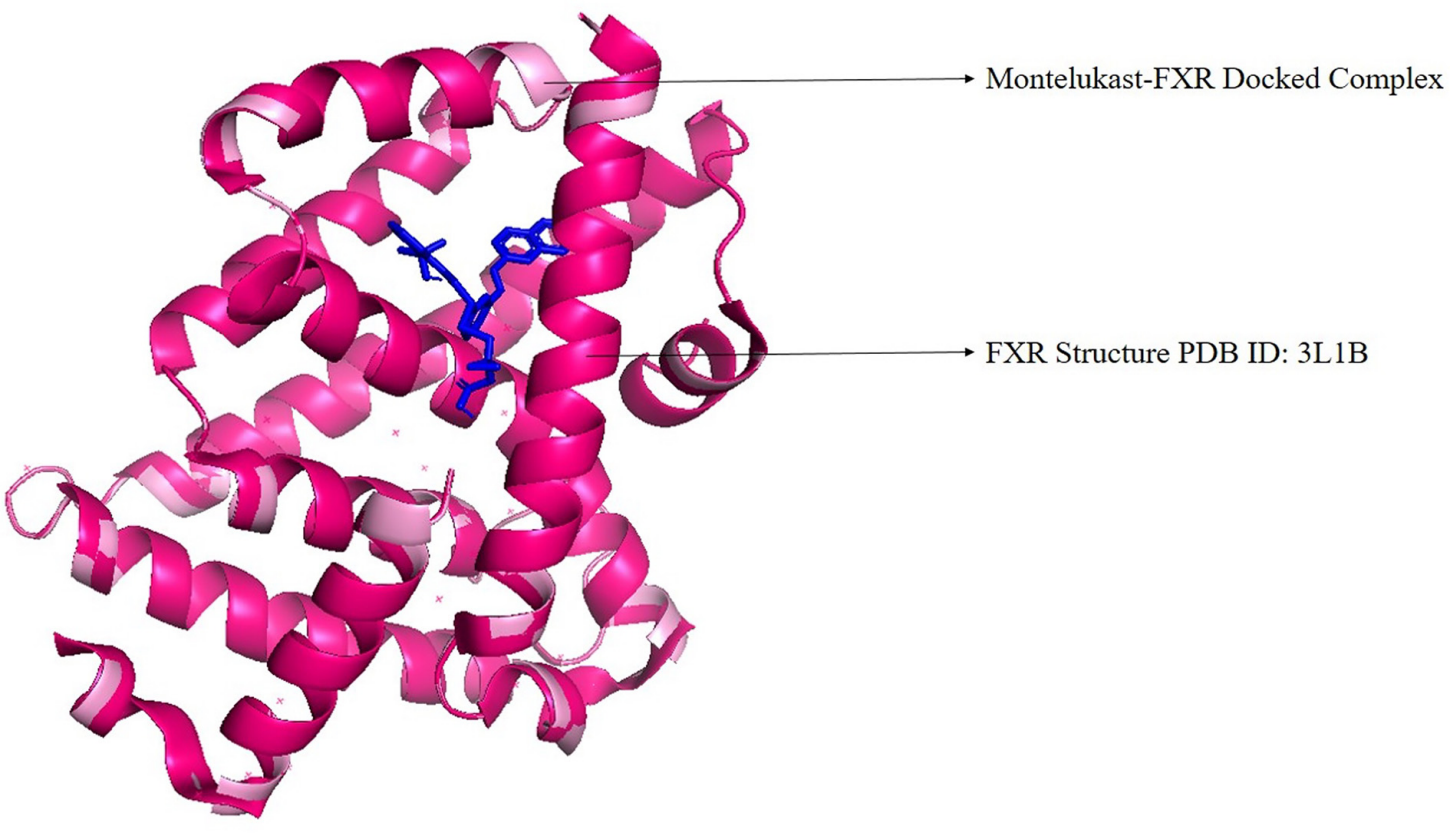
Superimposition of FXR structure FXR structure (PDB ID: 3L1B) before docking (in dark pink) and after docking (in light pink), which denotes the stability of the interactions and the protein structure. The image is prepared using PyMol2.0.

**Figure 4:**
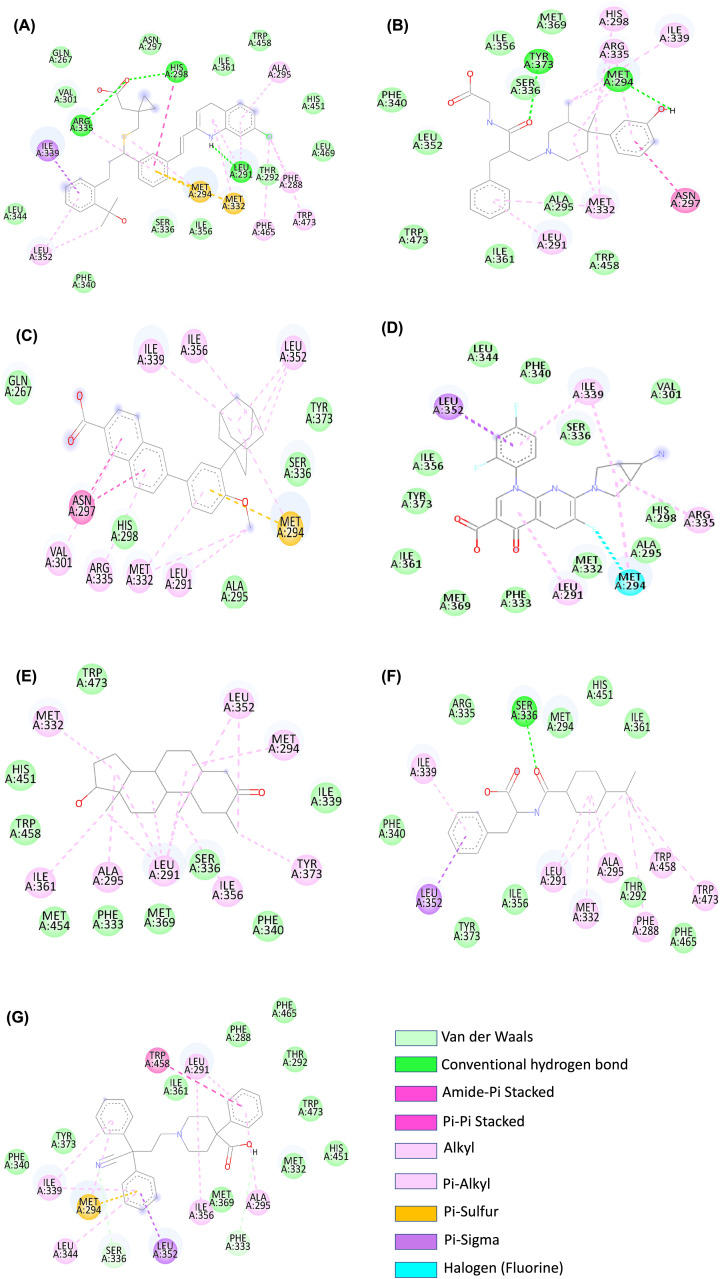
Amino acids of FXR showing various types of interactions with different drugs Two-dimensional representations of the binding site interactions of FXR with (**A**) montelukast, (**B**) alvimopan, (**C**) adapalene, (**D**) trovafloxacin, (**E**) drostanolone, (**F**) nateglinide, and (**G**) difenoxin namely, van der Waals, pi–pi stacked, pi–alkyl, conventional hydrogen bond, amide pi–stacked, pi–sulfur, alkyl, pi–sigma, pi–donor hydrogen bond, and carbon hydrogen bond. The interaction images are prepared using Discovery Studio3.5.

**Figure 5:**
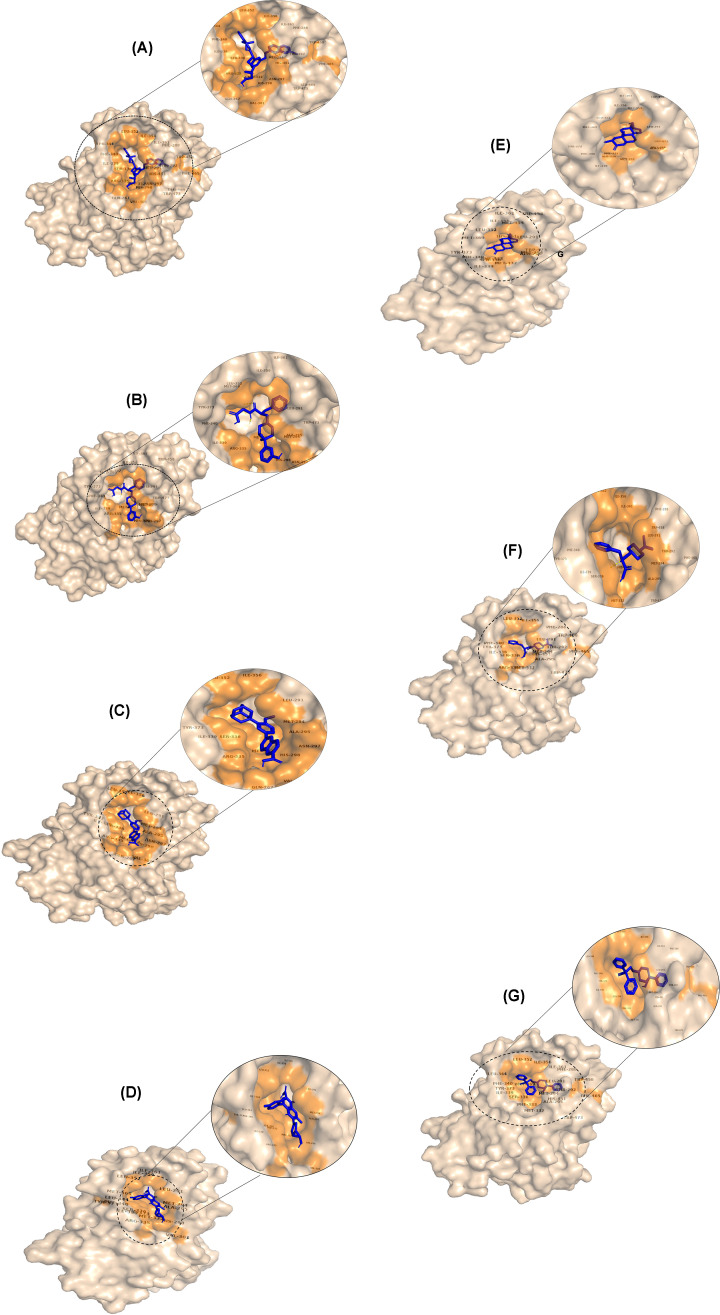
Three-dimensional conformation of the drugs in the interacting pocket of the FXR (**A**) Montelukast, (**B**) alvimopan, (**C**) adapalene, (**D**) trovafloxacin, (**E**) drostanolone, (**F**) nateglinide, and (**G**) difenoxin–protein complexes with the amino acids labeled displaying various types of interactions such as van der Waals, pi–pi stacked, pi–alkyl, conventional hydrogen bond, amide pi–stacked, pi–sulfur, alkyl, pi–sigma, pi–donor hydrogen bond, and carbon–hydrogen bond. The ligand is represented in blue and protein in tints-wheat while the interacting amino acids pocket is shown in orange color.

The major roles that FXR plays in the human body, and the diseases that can be regulated by the agonistic activity of this protein were represented in Figure 2. Examining the active site residues of the protein, the major amino acids that took part in the interaction were listed in the Table 1 and compared with other drugs under study. The molecular docking was performed using Autodock Vina to identify the most potent drug from the library. For each of the drug, nine docked conformations (total of 981 conformations) were obtained, and these were studied based on the binding affinity in terms of kcal/mol and RMSD values. The docking pose with the least RMSD value (zero value) was selected and examined for the interactions with the target protein. The docked protein–ligand complex was superimposed with the structure before docking and this revealed that complexes were stable. The control drug, obeticholic acid interacted with the protein with a binding affinity of only −7.6 kcal/mol. The RMSD value of superimposition was 0.000 for 1762 to 1762 atoms of FXR and FXR-montelukast complex respectively (Figure 3). Montelukast was shown to have the lowest binding energy of −10.3 kcal/mol with three hydrogen bonds and the highest affinity, while alvimopan was shown to have −9.1 kcal/mol with two hydrogen bonds. Other drugs showed higher levels of energy values indicating lower affinity between the drug and the protein. The ability to form complexes with the ligands is based on the binding affinity and the number of hydrogen bond interactions [65]. Further, the interactions of the drug and protein were stabilized by several low energy interactions such as van der Waal forces, alkyl bonds, pi–sigma, pi–cation, and amide bonds [66]. Montelukast with similarity score 0.006 exhibited three conventional hydrogen bonds with ARG 335, HIS 298, and LEU 291, van der waals interactions, pi–sigma, pi–sulfur, pi–pi stacked, amide–pi stacked, and alkyl interactions. Alvimopan with similarity score 0.039 exhibited only two conventional hydrogen bonds with TYR 373 and MET 294, van der waals, amide–pi stacked, alkyl, and pi–alkyl interactions. All the drugs with scores equal to or less than −8.4 kcal/mol, native ligand, and control drug were represented in the Table 1 with the major interactions taking place between the drug and the protein. Two-dimensional representation of the interacting amino acids with the drugs was shown in Figure 4, while Figure 5 showed the 3D representation of the drug in the interacting pocket of protein.

**Table 1:** Binding affinity of FXR in apo as well as in ligand-bound states consisting of types of interaction and interacting residues

Name of the compound	Type of interaction	Interacting residues	Binding affinity (kcal/mol)
Tetrahydroazepinoindole	van der waals	PRO 345, TRP 473, THR 292, PHE 465, PHE 288, HIS 451, TRP 458, ILE 361	
	Conventional hydrogen bond	SER 336, TYR 373	
	Pi–sigma	LEU 291	
	Pi–sulfur	MET 294	
	Alkyl, Pi–alkyl	LYS 343, LEU 344, LEU 352, ILE 339 (3), HIS 298, ARG 335, MET 369 (2), PHE 333, ILE 356, MET 332 (2), ALA 295 (2), MET 294 (2), SER 336, LEU 291 (2)	
Obeticholic acid (Control)	van der waals	VAL 475, LEU 455, HIS 451, ALA 452, TRP 473, VAL 329, GLU 330, ASN 448, ARG 399, LYS 325	−7.6
	Conventional hydrogen bond	ARG 459, ASP 474 (2), GLN 400	
	Unfavorable donor–donor	GLY 326	
	Alkyl	MET 456	
Montelukast	van der waals	GLN 267, ASN 297, VAL 301, LEU 344, PHE 340, ILE 361, TRP 458, HIS 451, LEU 469, THR 292, SER 336, ILE 356	−10.3
	Conventional hydrogen bond	ARG 335, HIS 298, LEU 291	
	Pi–sigma	ILE 339	
	Pi–sulfur	MET 294, MET 332	
	Pi–Pi stacked, amide-Pi stacked	HIS 298, THR 292	
	Alkyl, Pi–alkyl	LEU 352 (2), ALA 295, PHE288 (2), PHE 465, TRP 473, ARG 335, MET 294, MET 332, LEU 291 (2)	
Alvimopan	van der waals	PHE 340, LEU 352, TRP 473, ILE 361, ALA 295, TRP 458, ILE 356, MET 369, SER 336	−9.1
	Conventional hydrogen bond	TYR 373, MET 294	
	Amide–Pi stacked	ASN 297	
	Alkyl, Pi–alkyl	HIS 298, ILE 339, ARG 335 (2), MET 332 (3), LEU 291, ALA 295, MET 294 (3)	
Adapalene	van der waals	GLN 267, HIS 298, ALA 295, SER 336, TYR 373	−9.1
	Pi–sulfur	MET 294	
	Amide–Pi stacked	ASN 297 (2)	
	Alkyl, Pi–alkyl	MET 294, LEU 291, MET 332 (2), ARG 335, VAL 301, ILE 339, ILE 356, LEU 352 (3)	
Trovafloxacin	van der waals	ILE 361, MET 369, PHE 333, MET 332, ALA 295, HIS 298, TYR 373, ILE 356, LEU 344, PHE 340, SER 336, VAL 301	−9.0
	Pi–sigma	LEU 352	
	Alkyl, Pi–alkyl	ILE 339 (2), ARG 335, MET 294, LEU 291	
	Halogen (Fluorine)	MET 294	
Drostanolone	van der waals	TRP 473, HIS 451, TRP 458, MET 454, PHE 333, MET 369, SER 336, PHE 340, ILE 339	−8.6
	Alkyl, Pi–alkyl	MET 332, ILE 361, ALA 295, LEU 291 (4), ILE 356, TYR 373, MET 294, LEU 352 (2)	
Nateglinide	van der waals	PHE 340, TYR 373, ARG 335, ILE 356, MET 294, HIS 451, ILE 361, THR 292, PHE 465	−8.4
	Conventional hydrogen bond	SER 336	
	Pi–Sigma	LEU 352	
	Alkyl, Pi–alkyl	LEU 291 (2), ALA 295, MET 332, PHE 288, TRP 458, TRP 473, ILE 339	
Difenoxin	van der waals	PHE 340, TYR 373, ILE 361, PHE 288, PHE 465, THR 292, TRP 473, HIS 451, MET 332, MET 369	−8.4
	Carbon hydrogen bond, Pi-Donor hydrogen bond	SER 336, PHE 333	
	Pi–sigma	LEU 352	
	Pi–sulfur	MET 294	
	Pi–Pi stacked	TRP 458	
	Alkyl, Pi–alkyl	ILE 339 (2), MET 294, LEU 344, ILE 356, LEU 291 (2), ALA 295	

From the list of residues which take part in the interaction, it is evident that the grid box covered all the active residues and that the interactions were stable. When compared with the control drug, alvimopan, and montelukast displayed stronger and stable interactions with FXR. Although adapalene, difenoxin, and trovofloxacin reported the binding energy more than or equal to −9.1 kcal/mol, unlike alvimopan and montelukast, they did not exhibit any conventional hydrogen bonds.

### ADMET analysis of the drugs

The drugs with binding energy equal to or less than −8.4 kcal/mol were studied for their ADMET properties. Studying the lipophilicity, water solubility, pharmacokinetics, and medicinal chemistry properties of the drugs revealed that the drugs with good affinity and stable MD properties were having good ADME properties and medicinal chemistry as the compounds did not report any Brenk or Pan-Assay INterference compoundS (PAINS alerts) [67]. The potential of a drug to cross the blood–brain barrier (BBB), which separates brain tissues from substances percolating in the blood system, determines its efficiency [68]. Drugs or chemicals are pumped back into the intestinal lumen and hepatic system by permeability glycoprotein (Pgp), an ATP-dependent drug efflux pump widely disseminated and expressed as a receptor on intestinal wall [69,70]. Logarithm of skin permeation coefficient (log *K*p) of the substance in the stratum corneum, namely the rate of transdermal delivery through the epidermal layer, was originally used to assess the health risk posed by dermal absorption of compounds [71,72]. Montelukast with highest affinity toward the target, was having no BBB permeation and did not inhibit the Pgp substrate and the reported value of GI absorption was low. Reporting considerable levels of lipophilicity, the drug showed good levels of skin permeation [73]. While examining the toxic effects of the drug, it was predicted negative for *in vitro* Ames test in TA100 strain (with and without metabolic activation by rat liver homogenate). Additionally, it was also predicted that the drug showed no carcinogenicity or hERG inhibition (low risk) [74–76]. Montelukast and Alvimopan were the drugs that were verified through this *in silico* work as a potent agonist of FXR, and this was studied for its ADME properties and it reported no Lipinski, Ghose, Veber, Egan, and Muegge violations [77–82]. These drugs were also verified for high GI absorption and showed no BBB permeability. Additionally, alvimopan showed good solubility when compared with montelukast which was poorly soluble. The performed *in silico* toxicity assays showed negative results for carcinogenicity in mouse and low risk hERG inhibition for alvimopan while Ames test reported the drug as mutagen. However, the test predictions of *in vitro* Ames test result in TA100 strain with or without metabolic activation by rat liver homogenate showed no mutagenic effects. Tables 2 and 3 represented the results of ADME and toxicity analysis, respectively, for all the drugs with an affinity equal to or less than −8.4 kcal/mol.

**Table 2:** ADMET and drug likeness evaluation of the drugs

Name of the Drug	Montelukast	Alvimopan	Adapalene	Trovafloxacin	Drostanolone	Nateglinide	Difenoxin
TPSA	95.72	89.87	46.53	101.45	37.3	66.4	64.33
Ali class	Poorly soluble	Soluble	Poorly soluble	Soluble	Moderately soluble	Moderately soluble	Soluble
GI absorption	Low	High	High	High	High	High	High
BBB permeant	No	No	No	No	Yes	Yes	Yes
Pgp substrate	Yes	Yes	No	Yes	No	No	Yes
Log *K*p (cm/s)	−4.39	−7.7	−3.35	−8.62	−5.32	−5.35	−6.97
Lipinski violations	2	0	1	0	0	0	0
Ghose violations	4	0	1	0	0	0	0
Veber violations	1	0	0	0	0	0	0
Egan violations	1	0	1	0	0	0	0
Muegge violations	1	0	1	0	0	0	0

**Table 3:** Toxicity prediction of the drugs

Name of the drug	Montelukast	Alvimopan	Adapalene	Trovafloxacin	Drostanolone	Nateglinide	Difenoxin
Ames test	Mutagen	Mutagen	Mutagen	Mutagen	Non-mutagen	Non-mutagen	Mutagen
Mouse carcinogenicity	Negative	Negative	Negative	Positive	Negative	Negative	Negative
hERG inhibition	Low risk	Low risk	Medium risk	Medium risk	Low risk	Medium risk	Low risk
*In vitro* Ames test results in TA100 strain (metabolic activation by rat liver homogenate)	Negative	Negative	Negative	Negative	Negative	Negative	Negative
*In vitro* Ames test results in TA100 strain (no metabolic activation)	Negative	Negative	Negative	Negative	Negative	Negative	Negative
*In vitro* Ames test results in TA1535 strain (metabolic activation by rat liver homogenate)	Negative	Positive	Negative	Negative	Negative	Negative	Positive
*In vitro* Ames test results in TA1535 strain (no metabolic activation)	Positive	Negative	Negative	Negative	Negative	Negative	Negative

## Molecular dynamic simulation

**Figure 6:**
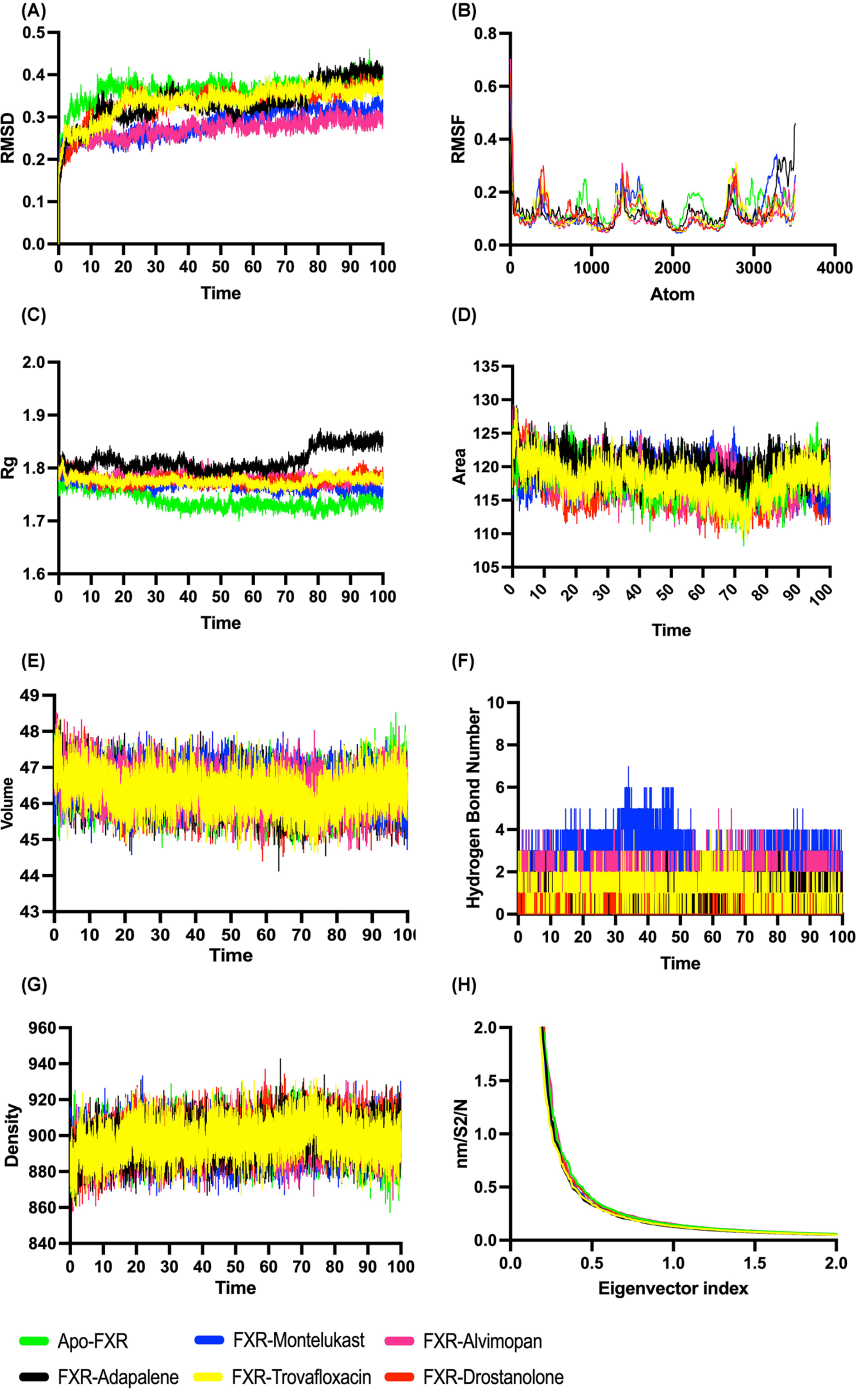
Molecular dynamics analysis on the FXR and FXR–drug complexes (**A**) The root mean square deviation plots, (**B**) root mean square fluctuations plots, (**C**) radius of gyration plots, (**D**) solvent accessible surface area analysis plots, (**E**) volume variations plots, (**F**) hydrogen bond analysis plots, (**G**) density variations plots, and (**H**) eigen vector index plots of the apo-FXR and potential compounds in complex with FXR.

When a ligand interacts to a protein-binding domain, the intended protein’s dynamics may encounter apparent folding modifications. The RMSD is among the most common primary characteristics for identifying whether a protein is resilient and near to the experimental structure [83]. The RMSD plot manifested that alvimopan and montelukast binding considerably stabilized the FXR protein which resulted in higher stability than the native protein with lower RMSD. This indicated that the fluctuations in the protein were lowered demonstrating its protein stabilizing activity. The average value of RMSD for alvimopan was 0.271985, fluctuating gradually from 0.0005027 to 0.3439814. Similar results were also observed for the drug, montelukast in which the average RMSD was 0.284203 while the fluctuation was observed between 0.0004951 and 0.358824. Interestingly, the native protein had RMSD values ranging from 0.0005043 to 0.4610075 and the average value of RMSD was 0.36466233, higher than alvimopan and montelukast. These results supported that the drugs which were chosen on the basis of structural similarity to obeticholic acid might be exhibiting stimulatory effect by stabilizing the conformations of the protein during the simulation of 100 ns. The binding of alvimopan to the binding cavity in the protein presented early minor fluctuations until 10 ns of the MD trajectories, after which it attained a RMSD value and remained stable from 10 to 100 ns. If we look at alvimopan’s dynamic behavior inside the protein-binding pocket, we can find that its dynamics were more stable and less fluctuating than the dynamics of the protein’s native structure (Figure 6A). Montelukast, on the other hand showed fluctuations until 45 ns and then attained lower deviations from the mean value till the completion of the simulation. As a result, the backbone of the protein became more stable during the 100 ns MD simulation.

RMSF of the protein’s backbone atoms was depicted in Figure 6B to investigate the dynamics of the protein’s backbone residues in protein–ligand complexes compared with the apo form of the protein. Figure 6B illustrated the RMSF of the protein’s backbone atoms, which were used to correlate the dynamic behavior and motion of the backbone residues in protein–ligand complexes to the apo form of the molecule per atom of the protein. The RMSF values of alvimopan and montelukast when in complex with protein were curtailed than the RMSF of the apo form of protein. As a result of the RMSF variation, alvimopan and montelukast stabilized the protein, indicating that it is more effective as an agonist. Alvimopan showed variations in RMSF between 0.0471 and 0.703 with lowest mean RMSF value, 0.112646605 when compared with the native protein (0.139965741), montelukast (0.122029784), and others. Montelukast displayed fluctuations in RMSF between 0.0444 and 0.6231 while native apo-protein showed fluctuations between 0.0581 and 0.6106.

### Radius of gyration

The concision of polypeptide during the time-frame of MD is measured by the *R*_g_ component. It is evident that it is employed to compute the distance between the protein’s center of mass and its endpoint in a specified time step. Overall, a well-functioning and conformationally stable protein has a reduced *R*_g_ value variation, which really is a crucial factor in determining stability analysis [84]. In this present work, the *R*_g_ values of the protein were illustrated in Figure 6C. The *R*_g_ results showed that compactness of FXR–alvimopan and FXR–montelukast complex were comparable to the apo-protein. The average value of *R*_g_ for alvimopan was 1.784194096 and it varied from 1.7524 to 1.82945 while montelukast ranged from 1.73845 to 1.81412 averaging to 1.768154597. The *R*_g_ values of the apo-protein averaged 1.739407876 while it varied from 1.69936 to 1.79382. This displayed the stability of the complex compared with the native protein.

### Hydrogen bond analysis

The hydrogen bond analysis is a significant component to examine when discussing receptor–ligand solidity since these interactions are an ephemeral contact that is accountable for the receptor–ligand complex’s stability [85]. We estimated hydrogen bonds for all of the complexes in this study since these are the main stabilizing interaction factor between two molecules. Figure 6F demonstrated the number of intermolecular hydrogen bonding at multiple time points that have been assessed. The number of hydrogen bonds estimated for FXR–montelukast and FXR–alvimopan were 0–7 and 0–5, respectively. The FXR–montelukast and FXR–alvimopan complex presented the highest number of hydrogen bonds, and hence it could be confirmed that the complex formed firm interactions. Thus, montelukast and alvimopan are the finest superior for drug considering extremely specialized hydrogen bond into account. Hydrogen bonds displayed were comparatively lower for other drugs such as adapalene, drostanolone, and trovafloxacin with 2, 3, and 4 hydrogen bonds each.

### Principal component analysis (PCA)

**Figure 7:**
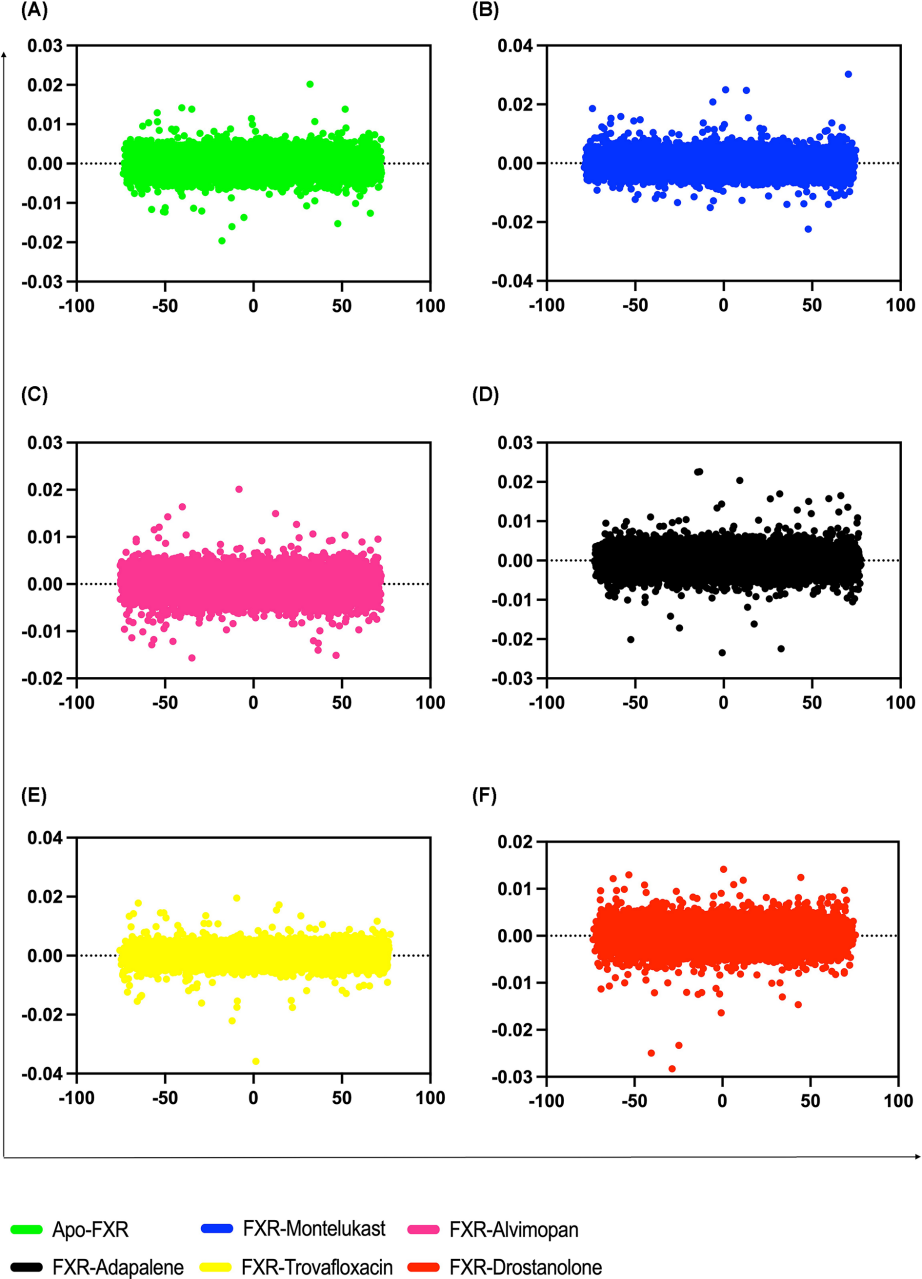
The principal component analysis of FXR and different drug complexes With the FXR backbone as the reference, PC1 and PC2 are plotted against each other of which (**A**) apo-FXR, (**B**) FXR–montelukast, (**C**) FXR–alvimopan, (**D**) FXR–adapalene, (**E**) FXR–trovafloxacin, and (**F**) FXR–drostanolone.

The overall pattern of motion of the complexes and apo-protein can be studied and the dynamics of the complex can be achieved by generating 2D projection plot in PCA. The very first two eigen vectors were found to encompass the entirety of the protein motions in all of the systems, suggesting that these vectors characterize the system’s important subspace [86]. Hence, the projection on the first (PC1) and second (PC2) principal components were used to gain a better understanding of the conformational changes in the FXR. The eigen values represented the atomic influence on the movement, whereas the eigen vectors defined the collective motion of the atoms [87]. FXR–montelukast and FXR–alvimopan complex show relatively higher space coverage and space magnitudes ranging from 74.64382 to −78.82188 and 72.2044 to −75.37371, respectively, throughout the simulation (Figure 7).

The trace values, which are the sums of the eigen values, for alvimopan and montelukast were 2801.880674 nm and 2799.553072 nm, respectively, while the sum of eigen value for apo-protein is 2864.35 nm (Figure 6H). It was suggested that apo-protein and protein–alvimopan appeared to cover a larger conformational space due to its greater flexibility when compared with the montelukast–FXR complex. This observation is in parallel with parameters taken for trajectory analysis such as RMSD, RMSF, and *R*_g_ of apo-protein and drug complexes which indicated the agonistic activity. When compared with the apo-protein and protein-alvimopan, the PCA plot showed that the cumulative motions of the protein–montelukast were contained in a restricted group. According to the anticipated flow of the research work, the FXR–alvimopan was more dynamic and stable throughout the 100 ns of simulation run.

### Solvent accessible surface area

SASA measures the interaction between complexes and solvents. SASA of the drug–protein complexes was calculated for predicting the extent of the conformational changes that occurred during the interaction [88]. Figure 6D showed the plot of SASA value versus time for the protein–drug complexes and apo-protein. The average SASA value for montelukast–FXR complex was 118.717 nm^2^ and FXR–alvimopan complex was 118.205 nm^2^ while the average SASA for the apo-protein was 118.200 nm^2^. These calculations showed that all the complexes had a significantly similar value of SASA as the apo-protein. Hence, in the present study, we observed that the SASA values for these two protein–drug complexes during 100 ns MD simulation were relatively stable, indicating no significant changes in the overall protein structure (structural integrity is maintained). The average density of the montelukast–FXR complex, FXR–alvimopan complex, and the apo-protein were 895.377, 896.124, and 897.062, respectively (Figure 6E), while the volume averages for the montelukast–FXR complex, FXR–alvimopan complex, and the apo-protein were 46.466, 46.428, and 46.379, respectively (Figure 6G).

## Discussion

In the present work, rational approach combining receptor-based virtual screening and MD simulations have been employed on the target FXR to identify potential activators. A focused library of 110 compounds based on the structural similarity to obeticholic acid, which is an FDA-approved drug currently used as FXR agonist was prepared. FXR is highly expressed in both the liver and the intestine. Both hepatic- and intestinal-FXR are involved in the regulation of bile acid homeostasis [89–91]. FXR is a nuclear hormone receptor with high expression in liver. It regulates many genes involved in lipid and glucose metabolism, liver regeneration, inflammation, and liver cancer. FXR knockout mice showed sustained activation of the Wnt/β-catenin pathway, in which the tumor suppressor gene, N-myc downstream-regulated gene 2 was reduced, and spontaneously develop tumor [89,92,93]. Hence, *in vitro* and *in vivo* experiments showed that FXR agonists have a vital role in suppressing cancer causing oncogenes and in regulating conditions such as proteinuria, glomerular inflammation, and fibrosis, and therefore, targeting FXR for developing therapeutics is a potential area of research. Many *in vitro* studies have been used to identify potential FXR agonists and in this work, we used the resolved FXR structure to find suitable compounds at a quicker pace [19,32,94,95].

Molecular docking studies have revealed that seven compounds screened possess strong interaction with higher binding affinities. We observed that their docking scores of top complexes ranging from −8.4 to −10.3 kcal/mol. In this research, we focused on the top docking results for further analysis as these drugs show higher binding affinity and share the same binding pocket with similar interacting amino acid residues of FXR protein. FXR shares a common nuclear receptor organization consisting of a ligand independent and dependent activation domain, DNA-binding domain, and hinge region [96]. The FXR–ligand binding domain interacts with its ligands, and it constitutes 12α helical domains which is present in the form of a helical sandwich. This domain contains a hydrophobic ligand binding core, which accommodates the ligands and consists of AF2 and H12 domains [97]. Highly hydrophobic amino acids such as LEU 352, LEU 291, MET 332, ALA 295, PHE 465, TRP 473, and ILE 339 are present in this domain which forms hydrophobic interactions to the FXR-agonists, such as montelukast and alvimopan. Polar bonds are formed between the drugs and critical amino acid residues such as LEU 291, HIS 298, MET 294, ARG 335, and TYR 373 which stabilizes the structure of the docked complexes. The high number of interactions between these residues suggests that they play key roles in the binding of compounds. When compared with other drugs, such as adapalene, difenoxin, drostanolone, nateglinide, alvimopan, and montelukast are highly interacting drugs with FXR and forms stable complex during molecular docking forming conventional hydrogen bonds with the protein. These results of molecular docking were proven accurate with the results of MD simulations.

A 100 ns MD simulation was performed for the drug–protein complex and the apo-protein in order to identify and evaluate the stability of the docking processes that was carried out. The RMSD plot proposes that both the drugs are stable throughout the dynamic simulations and RMSD values did not display any rapid upwelling or slithering throughout the simulations. FXR–montelukast and FXR–alvimopan complexes have similar RMSD values showing lesser deviations. RMSD plot reveals that apo-protein has considerable stability than RMSD plots of FXR–montelukast and FXR–alvimopan complexes. The stable *R*_g_ plot of FXR implies the maintenance of the overall structural integrity of FXR during the due course of simulation length. The *R*_g_ of the apo-protein and drug complexes was found to be similar for all the complexes. Next, the Rg variation of FXR–drug complexes also appears to fluctuate in a similar range 1.7–1.9. RMSF was calculated for FXR with 3511 atoms and five potential drugs. It provides evidence concerning the solidity of the complex and the ability of the drugs to stabilize the protein steadiness. Lower the number of fluctuations from the mean value expresses more stable bonds, supporting agonistic activity [98]. The average RMSF value for the complex was 0.122 nm for FXR–montelukast complex and 0.112 nm for FXR–alvimopan complex while higher was the average RMSF (0.139 nm) of the apo-protein. To additionally explain the stimulatory potentiality of the drugs, hydrogen bond analysis was performed along with PCA and SASA calculations. These indicated that the protein and drug complex shows resilient bonding contacts throughout the 100 ns of the MD simulations. To investigate the atomic motions throughout drug binding in both the apo-protein and protein complexes, the PCA was performed from the trajectories. The PCA results specified that all the drug complexes show firmness in space coverage, which allude to radical steadiness seen over the protein PCA result [86]. SASA finds the area of receptor exposed to the solvents throughout the dynamic simulation procedure [99]. We found the average SASA value of the protein as 118.2 nm^2^. The subjection of the hydrophobic portion of receptor protein residues due to the binding of the drug molecule toward receptor constantly add to SASA value.

The ADMET characteristics are important in drug discovery and development [100]. Physicochemical and pharmacodynamics properties of the drugs such as topological polar surface area (TPSA), GI absorption, solubility class, and BBB permeation are important features that contribute to efficacy and safety [101]. Accordingly, it is essential to discover efficacious molecules with improved ADMET properties. For optimized formulation applications in pharmaceutical research, solubility and GI absorption are critical [102,103]. Alvimopan reported good levels of GI absorption, solubility while it was predicted to possess no BBB permeation and the drug can act as a substrate for Pgp [104–107]. A molecule with a lower negative log *K*p value is seen as being more permeant to the skin (−8.62, less permeant; −3.35, more permeant). Although adapalene was reported to be most skin permeant, alvimopan reported considerably good levels of permeation.

Hence, montelukast and alvimopan are the potential drugs as FXR agonists from the present study. Currently, montelukast is used for asthma complications and alvimopan is used as an opioid antagonist to diminish the healing time of the GI tract following surgical interventions [108,109]. However, the effect of the drug in modulating the function of FXR such as for the treatment of ulcerative colitis, cancer, and renal lipid accumulation was never discovered. In the present work, the conclusion can be made that FXR–montelukast and FXR–alvimopan complex are very strong and that these drugs have considerable potential to stimulate the protein functionality. Moreover, in view of the fact that the present work is focused on a library of FDA-approved drugs, the safety of the drugs for human use is well established in terms of pharmacodynamics, bioavailability, and medicinal chemistry. The major limitation of the study is the lack of integration of *in silico* techniques with *in vitro* or *in vivo* experimental studies for lead optimization. Additionally, more precise and reliable post-processing methodologies, including free-energy methods based density functional theory (DFT) calculations, should be performed [110]. Further, we recommend that the drugs must additionally be validated and verified and their *in vitro* and *in vivo* agonistic prospective needs to be examined.

## Conclusion

In the context of chronic diseases like cancer, proteinuria, ulcerative colitis, and irritable bowel syndrome, the role of finding therapeutics is highly essential. In the present study, docking experiments were carried out on the FXR protein to find potential agonists of the same from a library of drugs created by assessing the structural similarity to the FDA-approved drug, obeticholic acid. Further, this was confirmed by MD simulation for 100 ns. The present study revealed drug repurposing potential of drugs including alvimopan and montelukast. The MD simulation and trajectory analysis revealed the stability of the protein–drug complex thus formed. Validation was done using several tools of GROMACS including SASA, hydrogen bond analysis, and PCA. However, these results should be experimentally verified using *in vitro* and *in vivo* experiments before proceeding for clinical trials.

## Summary

- FXR is a therapeutic target for the treatment of several diseases like glomerular inflammation, cancer, and tubulointerstitial fibrosis.
- Computer-aided drug discovery is a fast and robust way to accelerate the process of identifying potential drug candidates for several diseases with known targets.
- The drug candidates identified by this *in silico* study show good agonistic activity toward FXR.
- However, *in vitro* and *in vivo* studies are required to confirm their agonistic activity and binding affinity.

## Data Availability

The data presented in this article can be obtained from the corresponding authors upon reasonable request.

## Abbreviations

ADMET: absorption, distribution, metabolism, excretion, and toxicity
FDA: Food and Drugs Administration
FXR: farnesoid X receptor
GROMACS: GROningen MAchine for Chemical Simulations
LINCS: LINear Constraint Solver
MD: molecular dynamics
NASH: non-alcoholic liver steatohepatitis
PCA: principal component analysis
Rg: radius of gyration
RMSD: root mean square deviation
RMSF: root mean square fluctuations
RXR: retinoid X receptor
SASA: solvent accessible surface area
TPSA: topological polar surface area
UDCA: ursodeoxycholic acid
